# AVPR1A and Stress in Adults With Sickle Cell Disease–Related Chronic Pain

**DOI:** 10.1093/geroni/igaa057.2843

**Published:** 2020-12-16

**Authors:** Keesha Roach, Brenda Dyal, Srikar Chamala, Yingwei Yao, Roger Fillingim, Zajie Wang, Robert Molokie, Diana Wilkie

**Affiliations:** 1 University of Florida, Gainesville, Florida, United States; 2 University of Florida, Pain Research and Intervention Center of Excellence, Gainesville, Florida, United States; 3 University of Illinois at Chicago, Chicago, Illinois, United States

## Abstract

Purpose: Emotional stress is a known pain trigger in patients with sickle cell disease (SCD). The Arginine vasopressin receptor 1A gene (AVPR1A), SNP rs10877969, is associated with acute pain and stress-related pain. Our study investigated the association between AVPR1A genotype with stress and age in adults with SCD pain. Methods: 169 participants with SCD and chronic pain (100% African descent; mean age 36.4 ± 11.6 years [range =18-74 years]) completed the Perceived Stress Questionnaire. The SNP was evaluated as the imputed score was R2>0.8. ANOVA compared stress by genotype and age. Findings: Mean stress scores were significantly lower (p<0.05) for the older adults (0.35 ± 0.18) than the younger adults (0.41 ± 0.17). Mean stress scores were not significantly different by genotype for younger or older adults. Discussion: The rs10877969 genotype frequency was not different by age. In contrast to prior research, there was no association between genotype and stress.

